# Hierarchical Structure of Depression Knowledge Network and Co-word Analysis of Focus Areas

**DOI:** 10.3389/fpsyg.2022.920920

**Published:** 2022-05-19

**Authors:** Qingyue Yu, Zihao Wang, Zeyu Li, Xuejun Liu, Fredrick Oteng Agyeman, Xinxing Wang

**Affiliations:** ^1^College of Computer Science and Technology, Nanjing University of Aeronautics and Astronautics, Nanjing, China; ^2^College of Medicine, Jiangsu University, Zhenjiang, China; ^3^Jingjiang College of Jiangsu University, Zhenjiang, China; ^4^School of Management, Jiangsu University, Zhenjiang, China

**Keywords:** depression, knowledge network, hierarchical structure, word frequency statistical analysis, visualization network

## Abstract

Contemporarily, depression has become a common psychiatric disorder that influences people’s life quality and mental state. This study presents a systematic review analysis of depression based on a hierarchical structure approach. This research provides a rich theoretical foundation for understanding the hot spots, evolutionary trends, and future related research directions and offers further guidance for practice. This investigation contributes to knowledge by combining robust methodological software for analysis, including Citespace, Ucinet, and Pajek. This paper employed the bibliometric methodology to analyze 5,000 research articles concerning depression. This current research also employed the BibExcel software to bibliometrically measure the keywords of the selected articles and further conducted a co-word matrix analysis. Additionally, Pajek software was used to conduct a co-word network analysis to obtain a co-word network diagram of depression. Further, Ucinet software was utilized to calculate K-core values, degree centrality, and mediated centrality to better present the research hotspots, sort out the current status and reveal the research characteristics in the field of depression with valuable information and support for subsequent research. This research indicates that major depressive disorder, anxiety, and mental health had a high occurrence among adolescents and the aged. This present study provides policy recommendations for the government, non-governmental organizations and other philanthropic agencies to help furnish resources for treating and controlling depression orders.

## Introduction

Depression is described as a group of conditions connected with the lowering or elevation of an individual’s mood (Petry et al., 2008; Stetler and Miller, 2011). Depression is a common mental illness that seriously affects the quality of human life and even causes serious consequences such as self-harm and suicide (Rosenbaum et al., 2014; Huang and Zhao, 2020). Plausible causes of depression are traced to psychological, biological, and social sources of distress (Dunkel Schetter, 2011; Schetter and Tanner, 2012). Extant studies have demonstrated that these factors may result in a change in brain functioning comprising changes in activities of specific neural circuits in the human brain (Byrne et al., 2016; de Figueiredo et al., 2021). Depression is usually found in about 8–10% of the general population (Guillamondegui et al., 2011). Frequent feelings of sadness characterize depression, despair, worthlessness, recurrent inattention, fatigue, and loss of interest in favorite things, including adverse effects on appetite, weight, and sleep (Shah et al., 2014). Depression affects many aspects of life and can cause pain and suffering to individuals’ physical and mental health, social interactions, and work-life (Shah et al., 2014). Patients with depression often have to endure more intense physical illness and pain than in regular treatment and the ensuing decline in physical and social status and work capacity due to the disorder. Epidemiological data show that women are 70% more likely to suffer from depression than men (Shah et al., 2014). In addition, studies have shown that the frequency of depression increases with age (Morete et al., 2018). As early as 2017, the number of people with depression worldwide exceeded 300 million, according to the World Health Organization (WHO) (WHO Depression, 2017). Thus, psychological distress is a significant challenge in contemporary periods affecting adolescence and the aged, which merits in-depth investigations, interventions, and prevention measures (Lan and Wang, 2020). However, extant investigations ignore this crucial topic. This study is motivated by the impact of depression on individuals, especially the current COVID-19 depressive symptoms and its corresponding repercussions on individuals. Depression creates a feeling of loss of interest and sadness in engaging oneself in a series of activities that do not support the body’s normal functioning and create insecurity when exposed (Crockett et al., 2020).

Additionally, depression causes a series of physical and emotional issues and declines individuals’ propensity to work effectively and efficiently (Rosenbaum et al., 2014; Huang and Zhao, 2020; Lan and Wang, 2020). The frequency of the different kinds of psychopathology impacts individuals comprising anxiety and depressive disorders increases during the adolescent stages (Elsayed et al., 2019; Rapee et al., 2019). In reality, life transition is characterized by physical, psychological, and social changes, which creates adolescents to experience intense and frequent emotional trauma compared to adults and children (Crockett et al., 2020). Further, adolescents who have lost their relatives or experienced parental migration are challenged with additional psychological distress due to the absence of relatives or parents characterized by limited educational and social resources in diverse environments (Lan and Wang, 2020).

Studies have indicated that psychological adjustment and socio-ecological structures are ecological conditions established and perpetuated over time due to complex interactions between intra and inter-individual factors (Lan and Moscardino, 2019; Lan et al., 2019a; Lan and Wang, 2020). These conditions suggest that ecological system theories furnish a valuable structure for evaluating the safeguarding factors for depression-related symptoms in individuals or adolescents who might face the absence of their parents or relatives in development stages or crisis periods. Thus, depressive conditions among individuals in crisis periods are central to depression. A critical example is the impact of the COVID-19 on adolescents’ life, including schooling. At the same time, adults are also challenged with the mode to survive in the absence of jobs and restrictions on going to the workplace even if they have access to job opportunities (Ivanova and Israel, 2005; Lan and Moscardino, 2019; Lan et al., 2019a; Lan and Wang, 2020; Shi et al., 2020; Santomauro et al., 2021). The pandemic of Coronavirus disease 2019 (COVID-19) has also impacted people’s mental health (Huang and Zhao, 2020; Serafini et al., 2020; Wang et al., 2021). In a meta-analysis investigation conducted by Santomauro et al. (2021), it was estimated that the outbreak and spread of the COVID-19 pandemic and Newcastle disease virus had increased the number of cases of major depression by more than 50 million cases worldwide, with a growth rate of 28% (Shirvani and Samal, 2020). According to a German survey of a population of more than 5,000 adults, depressive symptoms increased by 14.3% (Bäuerle et al., 2020). Also, a study conducted by the German Association of Psychotherapists at the beginning of 2021 indicated that the number of consultations for psychological problems increased by 40% compared to previous years (Rabe-Menssen, 2021). When faced with a major catastrophic event, the public is guaranteed physical safety and experiences psychological shock or trauma. Studies have demonstrated that out of thousands of participants from 194 cities in China, 53.8% were unaware of the virus (Wang et al., 2020). Therefore, the participants rated the psychological impact of the epidemic based on the prevailing conditions. Thus, adverse emotional states and psychological problems such as stress, anxiety, depression, frustration, and anxiety gradually developed during the COVID-19 pandemic (Serafini et al., 2020). This study is premised on enormous depressive conditions associated with adolescents’ development and the adults that scholars have investigated. This study contributes to the literature by applying a hierarchical structure to a depression knowledge network and co-word analysis in focused jurisdictions. This paper employed the bibliometric methodology to analyze 5,000 research articles regarding depression. This current research also employed the BibExcel software to bibliometrically measure the keywords of the chosen articles and further conducted a co-word matrix analysis. Additionally, Pajek software was used to conduct a co-word network analysis to obtain a co-word network diagram of depression. Further, Ucinet software was utilized to calculate K-core values, degree centrality, and mediated centrality to better present the research hotspots, sort out the current status and demonstrate the research characteristics in the area of depression with crucial information and guide for successive investigations. Thus, this research found that few studies have been conducted and demonstrated that depression associated issues are worth in-depth investigations, interventional strategies, and prevention (Jeffcoate et al., 2018; Cooke et al., 2019; Rahmadiana et al., 2021; Sun et al., 2021). This study seeks to create awareness and bridge the study gap created in this crucial field. As depression now received more attention, the research on depression has become more intensive, and the scope is widening. To explore the current situation, this study contributes to knowledge by applying research hotspots and development trends in the field of depression. This research uses knowledge graph analysis to construct a network and visualize the literature on depression in China National Knowledge Infrastructure (CNKI) database to explore the characteristics of current research in this field and provide recommendations for future research.

## Review of Literature

Recent studies indicate that depression has been studied for a long time and continues to receive much attention from distinct researchers, especially in recent years (Jeffcoate et al., 2018; Cooke et al., 2019; Lan and Moscardino, 2019; Lan et al., 2019a; Rahmadiana et al., 2021; Sun et al., 2021). This study categorizes the diverse aspect and branches of depression analysis in the following contexts: factors triggering depression, depression-associated symptoms and disorders, and prevention and treatment of depression.

### Factors Triggering Depression

Depression is characterized as the change in mood of individuals that affects their daily lives and activities (Peeters et al., 2003; Kneeland et al., 2020). Efficient treatment, diagnosis, and support are the concrete path to assist individuals undergoing depression to recover (Rogers et al., 2001; Wichers et al., 2011; Bamelis et al., 2014). Depression is a medical condition that impacts individuals with no exception for race, gender, and income level (Chen et al., 1999; Assari, 2017). Individuals with medical depression mostly feel worried and hopeless and experience deep emotional pain for a prolonged period (Rogers et al., 2001; Assari, 2017). Depression is a complex disease whose precise cause is hardly determined. However, it may emerge from various reasons or sources, including the death of a relative, abuse, age, medication, conflicts, substance misuse, change in environment, distance from loved ones, and others (Azim and Baig, 2019; Schroder et al., 2020). Thus, a series of reasons cause individuals to endure depression. For instance, (Armour et al., 2014; Spinhoven et al., 2014) clarified that many adverse psychological reactions might be triggered after experiencing enormous traumatic conditions. Depression and post-traumatic stress disorder (PTSD) are the two types of traumatic disorders prevalent and significantly impacting individuals (Lai et al., 2013; Kukihara et al., 2014). In addition, the adolescence stage is also a critical factor, and it is a period when depression is dominant and determined among youth (Meurs et al., 2015; Rapee et al., 2019; Lan and Wang, 2020; Zhou et al., 2020). This period is quite challenging during the early years when adolescents’ emotional and physical characteristics develop rapidly, which increases the likelihood of depressive symptom onset in adolescents. Significant physical and psychological changes occur during adolescence, associated with increased emotional control and social skills. Nevertheless, it may also elevate susceptibility to depression (Calandri et al., 2019). Studies have shown that family and the immediate environment also strongly affect depression (Ivanova and Israel, 2005). The findings of Yan et al. (2021) shed light on the fact that family habits during childhood significantly affect people’s lives and depressive status. These revealed how family habits during childhood affect people’s depressive conditions. COVID-19 is highly contagious. It has led to a specific mortality rate, which leads to feelings of uneasiness, fear, and anxiety, leading to depression or other psychological disorders (Spoorthy et al., 2020).

### Depression Associated Symptoms and Disorders

The assessment of depression and its related symptoms relating to the biological and psychological issues of individuals is vital because of its accompanying negative thought content, suicidal notions, and cognitive dysfunction that may impact citizens’ socio-economic conditions (Hirsch et al., 2018; Watkins and Roberts, 2020; Kupfer, 2022). Depression has a wide range of symptoms and can have other comorbidities (Brailean et al., 2020; Gold et al., 2020). For example, people with obsessive-compulsive disorder and major depressive disorder have lower levels of competence in dealing with people, social interactions, and work (Moritz et al., 2018; El-slamon et al., 2022). People with depression also experience high anxiety levels, such as agitation, apprehension, and worry (Moore and Howell, 2017). Depression is related to strong negative emotions, including panic, anger, sadness, loneliness, self-blame, and resentment. At the same time, weak negative emotions include calmness and relaxation. These negative emotions elaborate on the different emotional manifestations of obsessive-compulsive disorder (OCD) and depression (Moore and Howell, 2017). and it is evident from many survey data that depression is diagnosed simultaneously as at least one-third of OCD patients are diagnosed (Nestadt et al., 2001). Similarly, most depression patients are usually accompanied by symptoms including obsessive-compulsive disorder (Sağlam et al., 2018; Guzick et al., 2020).

According to previous data, anxiety is one of the most prevalent problems worldwide, with more than 5% of the population suffering from excessive internal anxiety (Yu et al., 2018; Shi et al., 2020), and nearly 40% of those with anxiety disorders have reached the level of depression (Hunt et al., 2002). One-quarter to one-half of adolescents with primary depression is mainly diagnosed with anxiety disorders in previous studies, and 10–15% of adolescents diagnosed with anxiety disorders suffer from depression (Garber and Weersing, 2010; Cummings et al., 2014). In 2020, a cross-sectional study on Chinese adolescents concluded that the probability of comorbidity between depression and anxiety was nearly one-third (Zhou et al., 2020). Furthermore, patients with depression and chronic pain suffer more intense illness and have much higher medical costs than chronic pain patients without depression (Rayner et al., 2016). Also, comorbid psychological disorders interfere with the diagnosis of depression. The examination of this treatment can increase the complexity and difficulty of treatment, even leading to undiagnosed depression, which can also have a more significant impact on the patient’s will to take the treatment (Gordon et al., 2006; Guillamondegui et al., 2011). Depression can also be affected by hypertension, and hypertensive patients are more likely to suffer from depression. Depression has emerged as a key factor resulting in hypertension (Davidson et al., 2000; Meurs et al., 2015; Koh et al., 2016). Thus, patients with both conditions have lower health, quality of life, higher treatment costs, and a higher probability of death than those with only hypertension (Oganov et al., 2011; Scuteri et al., 2011; Tsartsalis et al., 2016). Studies have demonstrated that the number of college students suffering from depression is continuously increasing, which can have a negative impact on their mental and physical health, such as insomnia (Eller et al., 2006), a decline in grades (Ruz et al., 2018), self-harm and suicidal thoughts, addiction to electronics (Matar Boumosleh and Jaalouk, 2017) and alcohol abuse (Mushquash et al., 2013).

### Preventive Treatment of Depression

Recurring depression denotes a significant public health issue and has merited the in-depth search for a strategic and successful long-run treatment of persons who develop recurring stages of depression (Dryman and Heimberg, 2018; McDaid et al., 2019; Kar et al., 2021). Efficient and effective preventative treatment strategies demand prolonged pharmacological management for most people with repeated depression (Ormel et al., 2019; Volkow et al., 2019; Izquierdo et al., 2021; Kupfer, 2022). Most depressive disorders are recurrent and chronic and require adequate treatment. Extant studies have demonstrated the relevance of identifying and focusing on the rapid treatment of depression to avoid depression severity and suicidal acts (Ormel et al., 2019; Kupfer, 2022). Notwithstanding the current debate on the increasing pattern of depression among youth and the aged, depression is diagnosable and treatable over the individual’s life (Horackova et al., 2019; Herrman et al., 2022; Kupfer, 2022). Most studies assess depression chiefly through the traditional classical test theory (CTT) to develop questionnaires and evaluate approaches to effectively determine depression prevention (Turner et al., 2007; Broderick et al., 2016). These methods are not only effective but less expensive with higher benefits (Stuart et al., 2014). Also, depression prevention investigations are conducted by scholars through interviewing to assess depression (Lim et al., 2018; Levis et al., 2019; Bueno-Notivol et al., 2021). The probability of people suffering from depression is exceptionally motivated and elevated in the face of stress and catastrophic events such as COVID-19 (Lim et al., 2018; Bueno-Notivol et al., 2021). Thus, the spread of the New Coronavirus places a great deal of psychological stress on citizens and health workers on the front line (Khan et al., 2020; Trumello et al., 2020; Munawar and Choudhry, 2021). According to the findings of Feng and Yin (2021), gratitude can reduce depression among medical workers by mediating it with social support and social hope. In addition, adequate social support can create a sense of security for people to vent their internal negative emotions and stress, thus reducing depressive symptoms in individuals (Hobfoll, 2001; Hobfoll and Shirom, 2001; Lan and Wang, 2020). Also, the study by Lan et al. (2019b) similarly confirmed that social support could alleviate depression. Thus, the extent of the impact of depression on individuals has contributed to the enormous investigations on depression with varying methodologies. For instance, a research conducted by Tan et al. (2018) using a computerized adaptive test (CAT-depression) for depression prevention and treatment demonstrated a positive effect that will aid in depression control.

## Methodology

This study is premised on bibliometric methodology. Further, statistical and knowledge network analysis is conducted to analyze units such as authors, keywords, and citations.

### Keywords Frequency Analysis

Word frequency analysis of keywords is the statistics and analysis of the frequency of keywords in papers obtained under the subject term search. Research has demonstrated that the frequency word analysis method is the most widely used methodology for keyword analysis (He, 1999; Baker, 2004; Chen and Xiao, 2016). TopN or TopN% in the keyword list are selected as the high-frequency keywords for most studies. This study further employed and embedded some widely used scientific and technical text mining and visualization software, such as CiteSpace. In addition, word frequency thresholds were used to distinguish between high and low-frequency keywords. The advantage of this method over the TopN selection method is that it is efficient in obtaining keywords that meet the requirements. Thus, this study’s methodological section predominantly used VOSviewer analysis and visualization software. This study made the following types of methods estimations regarding the keyword word frequency thresholds.

(1) Estimation using the low-frequency formula proposed in Zipf’s (Anderson, 1955; Irmay, 1997) second law:

(1) $$\frac{I_{n}}{I_{1}}=\frac{2}{n\,\left(n+1\right)}$$

*I_1_* refers to the number of keywords with a word frequency of 1 and *I_n_* refers to the number of keywords with a word frequency of *n*.

(2) The high-low frequency word formula (Donohue, 1973) was used for estimation.

(2) $$T=\frac{\left(-1+\sqrt{1+8\,I_{1}}\right)}{2}$$

*T* is the high-low frequency word demarcation threshold, *I_1_* refers to the number of keywords with occurrence number 1.

(3) Employing a corpus-based calculation (Sun et al., 1999).

(3) $$T=\frac{1+\sqrt{1+4\,D}}{2}$$

*T* is the high-low frequency word demarcation threshold, and *D* is the vocabulary size in the analyzed corpus, which can be simplified $T=\sqrt{D}$ thus, if the corpus size is sufficiently large.

(4) Using Zipf’s formula to obtain the high-frequency subthreshold estimations.

(4) $$P_{n}\approx C\,\left(l\,n\,n+\gamma \right)$$

*P_n_* refers to the sum of the first n keyword word frequencies, and γ is the Euler constant. If the application of the formula requires the condition that the slope of the formula is 1 when *n* = *c*,which indicates the relative balance between the number of keywords and the rise in frequency.

(5) Calculation of high-frequency words using Price’s formula for high-frequency cited literature.

(5) $$M=0.749\,\sqrt{N_{m\,a\,x}}$$

*M* is the high-frequency word threshold, *N*_*max*_ refers to the maximum value of word frequency.

(6) The word frequencies of keywords are arranged in descending order, and *g* is the threshold of high-frequency words if and only if the sum of the word frequencies of the current g keywords is not less than *g*^2^, and the sum of the word frequencies of the keywords (*g* + 1) is less than (*g* + 1)^2^, which is expressed as follows:

(6) $$\sum_{i=1}^{g}f_{i}\geq g^{2}$$

and satisfies,


(7) $$\sum_{i=1}^{g1}f_{i}\leq {\left(g+1\right)}^{2}$$

*g* is the number of high-frequency words in reverse order, *f_i_* refers to the word frequency of the *i^th^*keyword.

### Co-word Analysis

The co-word analysis of knowledge units is based on the interconnection of each unit (He, 1999; Chen and Xiao, 2016). Thus, it reveals the content association of information and the co-word relationship implied by feature items and constructs the relationship between homogeneous or heterogeneous knowledge units, including network analysis method, multivariate statistical analysis method, and visualization chart. Co-word network analysis uses statistical techniques to calculate the number of occurrences of word pairs as keywords in a paper, thus obtaining a co-word matrix, which is converted into a keyword co-word network. This method mainly uses scientometrics, knowledge graph analysis, subject hotspots, and topic structure research analysis. This study’s analysis is focused on extracting keywords from the literature, and the knowledge units are used as modules. If two words appear together in a knowledge unit, it demonstrates a co-word relationship. The co-word frequencies of all keyword pairs are calculated to arrive at the final co-word matrix.

### Analysis of Essential Indicators of Network Nodes

The network analysis of the nodes in the network is an integral part of this research. The fundamental evaluation metrics applied in this study include degree centrality, intermediary centrality, proximity centrality, K-core analysis, and feature vector centrality. This research divides the network analysis into layers by calculating the K-core values. The nodes are also analyzed based on the two types of metrics, thus the degree centrality and intermediary centrality nodes.

#### Degree Centrality of Nodes

As a quantitative indicator, degree centrality is usually used to measure the position of each node in the network in social network analysis. The higher the centrality of a keyword, the more it proves associated with more keywords in the network. Degree centrality can be divided into two categories: the number of edges directly connected to the node, and the second is the sum of the weights of all edges that are directly connected to the node.

#### Mediated Centrality of Nodes

The intermediate centrality indicates the number of shortest paths through the node in a network. In a network, the greater the intermediary centrality of a node, the greater the node’s role in communication between other nodes, expressed as follows.


(8) $$C_{b}\;=\sum_{s\neq i\neq t}\frac{n_{st}^{i}}{g_{st}}$$

*g*_*st*_ refers to the number of shortest paths from node *s* to node *t*.$n_{st}^{i}$ denotes the number of shortest paths through node *i* among the shortest paths from node *s* to node *t*.

#### K-Core Value

K-core value analysis was first proposed by Seidman (Batagelj and Zaveršnik, 2011; Kong et al., 2019), which represents a graph region consisting of all nodes in a network with degree values greater than or equal to K. The calculation process is as follows: remove nodes and edges in the network with degree values lower than K until all remaining nodes have degree values greater than or equal to K. Take the values 1, 2…, n for K in turn, and finally, in the resulting subgraph of K-core, each vertex has at least K degrees and all vertices are connected to at least K other nodes in the subgraph. K-core is usually used to subgraph a graph by removing unimportant vertices and exposing the expected subgraph for further analysis, which assumes the core position in the graph. The higher the core degree, the smaller the subgraph, and the larger the core degree corresponding to that subgraph. The subgraph divided by the core degree assumes a more critical role in the original graph.

### Anatomy of Knowledge Network Hierarchy

**FIGURE 1:**
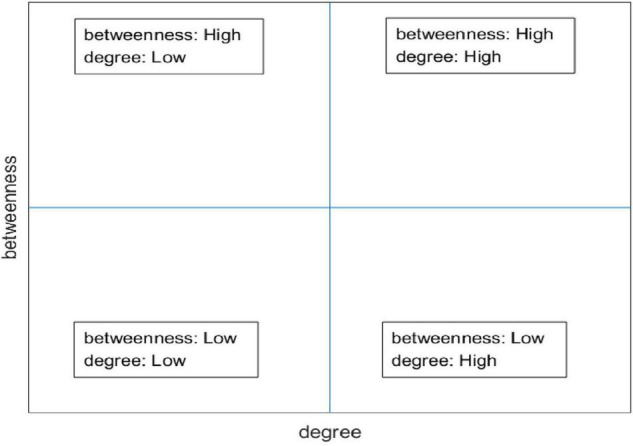
The node centrality analysis.

In this paper, different hierarchies were classified according to the K-core values. The positions between the different nodes and the meaning are determined by calculating the corresponding degree of centrality and intermediary centrality for the nodes in the hierarchy. Degree centrality is an essential local component to its neighboring nodes, and intermediary centrality is the widely intermediary component of a node to its non-neighboring points. The two-dimensional plan uses degree centrality and intermediary centrality as horizontal and vertical coordinates, as shown in Figure 1. The coordinate plane is equally divided into four regions. The upper right region indicates that both degree centrality and intermediary centrality are high, and their nodes have more significant influence in a local and global sense. The upper left region also indicates that degree centrality is low and intermediary centrality is high, while the node has greater influence globally. The lower left region indicates that both centrality and intermediary centrality are low, and their node has little impact locally and globally. The lower right region indicates high and low intermediary centrality, and their nodes have more influence in the local domain.

## Data Description

**FIGURE 2:**
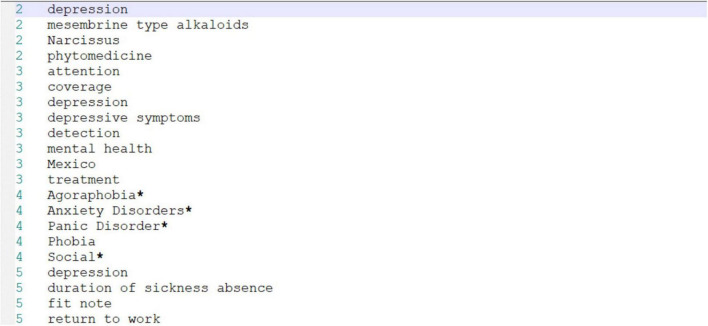
Development of paper numbers and keywords as marker fields.

Depression is a common mental illness that has an enormous impact on patients’ physical health, mental health, and even life. Studies on the prevention and treatment of depression in China are scarce. The adverse effects of depression in different age groups and populations are becoming more and more significant with the changing times. In this study, depression is employed as the research object, and the study data were gathered from the database of CNKI for analysis. This analysis began from 2008 through 2021. A total of 6,000 scientific indexing papers were retrieved, from which 5,000 papers were selected as samples for analysis, exported in Refworks format, and stored in TXT files. Further, the TXT file was imported into CiteSpace format conversion and processed through BibExcel, as shown in Figure 2. The first column in Figure 2 depicts the paper number, and the second column denotes the keywords in the paper.

After sorting a total of 7,508 keywords, 5,000 valid document keywords with a cumulative frequency of occurrence greater than or equal to 40 were extracted as high-frequency keywords based on the proportion of keywords with different frequencies in the thesaurus, totaling 29 keywords. A keyword co-occurrence matrix is constructed based on the word frequency threshold for the data that meet the threshold requirement. This co-occurrence matrix is imported into Ucinet for further analysis. In addition, a network file that can be applied to Pajek analysis was also generated based on the previous process file, thus preparing the visualization analysis for further analysis.

## Empirical Analysis

### Keyword Frequency Network Analysis and Visualization

**FIGURE 3:**
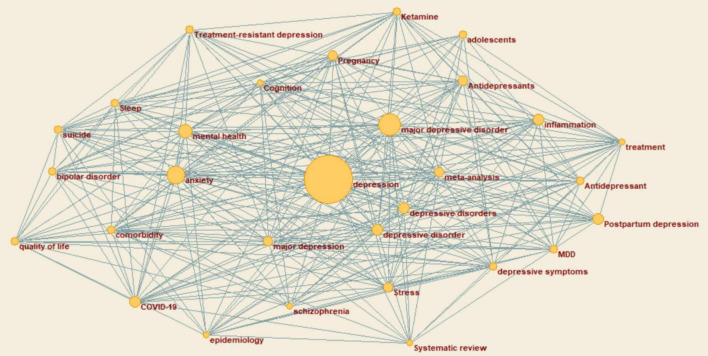
Pajek analyzes the results of network files and vector files.

The network files obtained from the above steps are put into Pajek by combining the vector files extracted from the frequency files. As shown in Figure 3, the plotted results where the vector files are assigned with the corresponding values to the different vertices in the plot.

Figure 3 indicates that the larger the area of the vertices, the higher the frequency of the keywords they represent, i.e., the more likely the term appears as a keyword under the theme of “depression.” In this paper, the keywords are divided into three echelons according to the word frequency, as shown in Table 1. Each echelon is selected to demonstrate the relatively representative keywords. The second layer includes major depressive disorder, anxiety, and mental health, which often account for a more significant proportion of depression. In addition, some words such as suicide, postpartum depression, and stress are relatively less frequent in the sample but are still inextricably linked to depression. In addition, the words between different echelons can also be related to each other. For instance, major depressive disorder and adolescents are connected in two nodes, indicating that the two can be used together as a direction of depression research, expanding more paths for the field.

**TABLE 1:** Distribution of keywords in different orders.

Number of steps	Keywords
1	Depression
2	Major depressive disorder, anxiety, and mental health
3	Suicide, postpartum depression, stress, treatment, and quality of life

### Depression Knowledge Network Hierarchy Classification

**FIGURE 4:**
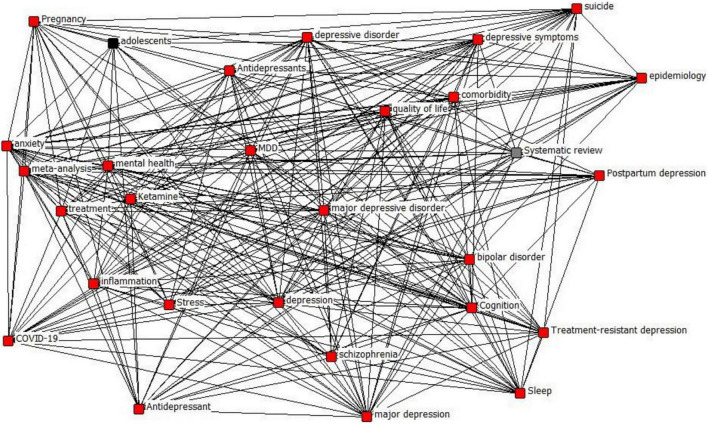
Node K-core value stratification.

The co-word matrix obtained above is imported into Ucinet and Netdraw. The degree of centrality, mediated centrality, and K-core values of nodes are calculated to reveal the complexity and core level of network relationships. First, the K-core values are calculated as shown in Table 2, and the corresponding images are drawn according to Table 2 and Figure 4. The K-core values obtained in this study have three categories, 12, 14, and 15, and have been distinguished by different colors, in which the sub-layer with a K-core value of 15 contains most nodes, the sublayer K-core contains 12 nodes. The sub-layer with a K-core value of 15 contains most of the nodes, the sub-layer with a K-core value of 12 contains adolescents, and the sub-layer with a K-core value of 14 contains a systematic review. This paper divides the knowledge network of depression topic words into three levels according to the K-core value. The first level has a K-core value of 12, the second level has a K-core value of 14, and the third level has a K-core value of 15. It can be concluded from the nodes of each level that the relevance of its nodes to depression topic words gradually increases in each level from low to high, and the scope also involved increases.

**TABLE 2:** K-core values for each node.

I.D.	*K-core
Anxiety	15
Depression	15
COVID-19	15
Mental health	15
Inflammation	15
Meta-analysis	15
Stress	15
Major depressive disorder	15
Epidemiology	15
Treatment	15
Adolescents	12
Comorbidity	15
Quality of life	15
Pregnancy	15
Sleep	15
Antidepressants	15
Systematic review	14
MDD	15
Depressive disorders	15
Ketamine	15
Suicide	15
Antidepressant	15
Cognition	15
Bipolar disorder	15
Schizophrenia	15
Depressive symptoms	15
Treatment-resistant depression	15
Postpartum depression	15
Major depression	15

Layers 1 and 2 contain adolescents and systematic review, respectively, which are relatively few words involved in the study of depression, while Layer 3 contains anxiety, stress, major depressive disorder, suicide, and antidepressants, which are closely related to depression and are the factors that researchers in this field have paid close attention.

### Depression Knowledge Network Node Analysis

**FIGURE 5:**
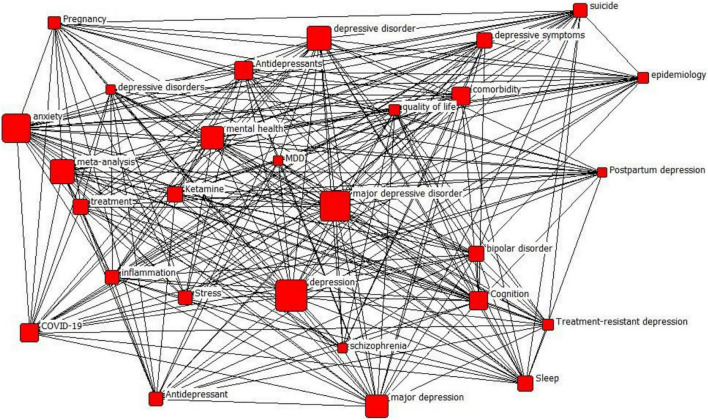
Degree centrality of nodes in the third layer.

**FIGURE 6:**
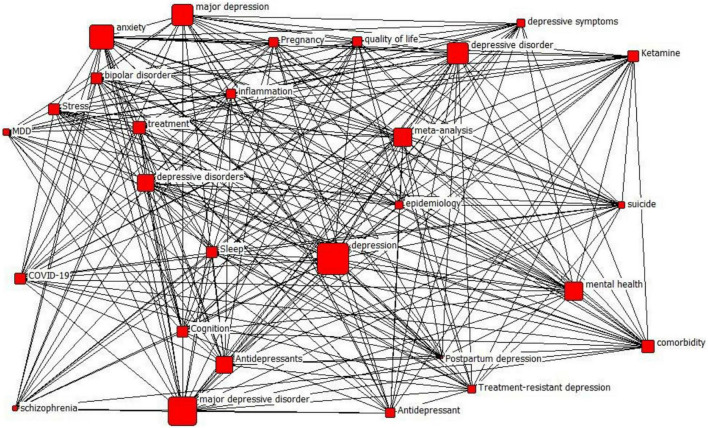
Mediated centrality of layer 3 nodes.

By calculating the degree of centrality and mediated centrality of nodes in different tiers, it is possible to determine the weight and role of the node among the layers. Centrality is a key reference coefficient to measure the degree of centrality of the whole network. In the network, the node in the central position acts as a bridge through which the rest of the nodes can connect with each other, and it has more power and a more robust influence on different nodes. This paper combines the degree centrality and intermediary centrality for analysis. Since there is only one node in both K-core values for layer 1 and layer 2, the degree centrality and intermediary centrality of different nodes are calculated for layer 3, as shown in Figures 5, 6. The larger the node, the greater the degree of centrality or intermediary centrality of the node. In other words, the greater the node’s role in constructing the network and connecting other nodes for communication.

**FIGURE 7:**
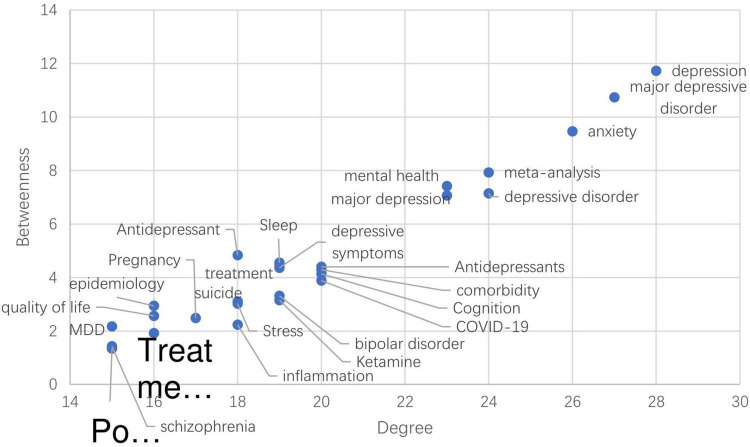
Relationship between two types of centrality distribution of each node.

Further, this study analyzes the interrelationship between the degree centrality of each node as the horizontal coordinate and the intermediary centrality as the vertical coordinate, as indicated in Figure 7. In the network analysis of layer 3, the degree of centrality and mediated centrality of depression demonstrated that the major depressive disorder and anxiety are relatively high. This indicates the importance of the three nodes in both the local and global networks. The degree of centrality of meta-analysis, depression, and anxiety are relatively high. The findings demonstrate that the meta-analysis, depressive disorder, mental health, major depression, antidepressants, comorbidity, Cognition, COVID -19, sleep, and depressive symptoms have high degree centrality and low mediator centrality. It indicates that these nodes are essential in a local network but do not play many roles in the overall network and the remaining nodes, including bipolar disorder. The remaining nodes, including bipolar disorder, ketamine, and epidemiology, have a relatively low degree of centrality and mediated centrality, which indicates that these nodes are not in a critical position in the intermediary of the layer 3 networks.

## Conclusion

This current study is based on the recent escalation of depression among individuals in China based on a rigorous analysis of previous research selected from the CNKI database. This study contributes to knowledge by combining robust methodological software for analysis, including Citespace, Ucinet, and Pajek. This study retrieved 5,000 articles with depression as the subject or keyword and constructed the knowledge network for analysis. This study draws the following salient conclusions:

(1) Taking the threshold value of 40 as the dividing line between high and low-frequency words, 29 high-frequency keywords were obtained, among which depression is the most occurring related keyword. In addition, major depressive disorder, anxiety, and mental health also had a high degree of occurrence. These high-frequency words contain the symptoms of depression, treatment directions, and others, which reflect the objective of this study and the current research on depression.
(2) The network was divided into three layers based on the calculated K-core values. This paper focused on the third layer of adolescents and systematic review, including depression, depressive disorders, depressive symptoms, and depression. This study found that in recent times COVID-19, stress, quality of life, getting treatment from hospitals, antidepressants, and ketamine are the significant factors influencing depression. Thus, the major areas are the research directions and branches meriting depression analysis. In addition, although adolescent depression has received a certain degree of attention in recent years, it still needs to be invested with more outstanding efforts in terms of the knowledge network structure of depression.
(3) This study measures the degree and mediated centralities of nodes. Further, this study analyzed the position and significance of nodes from global and local perspectives. This research helps determine the trend of current analysis on depression, major depressive disorder, and anxiety. This study contributes to the knowledge network of depression analysis. It further described the connection of different pairs of words, including high-frequency keywords and associations between other nodes. This research deepened the network structure and thus expanded the scope of the depression research field.

### Limitation and Future Directions

This study presents a systematic review analysis of depression based on a hierarchical structure approach. This research provides a rich theoretical foundation for understanding the hot spots, evolutionary trends, and future related research directions and offers further guidance for practice. Notwithstanding, this study has limitations as most studies. One of the salient limitations that characterize this research is based on methodology, sample selection, and screening processes. This study was premised on data collected from the Chinese National Knowledge Infrastructure (CNKI) database, which should have been extended to global sources of article retrieval, including the Web of Science. Future studies are projected to be conducted using data from the Web of Science for analysis with a corresponding increase in the samples selected and a rigorous screening process to be employed than what we applied recently. Although the methodologies and softwares used are current and produce accurate results, future studies are targeted to improve these approaches. The authors have further planned to conduct another investigation blending qualitative and quantitative methods to analyze the depression disorder issues from a global point of view.

### Theoretical and Practical Contributions

By comparing the current prevalence and impact of depression disorder among individuals in recent years (Meurs et al., 2015; Rapee et al., 2019; Lan and Wang, 2020; Zhou et al., 2020), this study suggests that this phenomenon should be considered a critical problem that merits in-depth investigation and modeling. Also, it is crucial to determine appropriate methodologies for diagnosing individuals who might face depression disorder issues or those who have been diagnosed and need enormous attention and interventional measures to prevent its prolonged effect on the patients. Based on the studies analyzed, this study found that most governments have not paid much attention to providing resources for the various facilities that assist patients with depression disorder to enhance effective and efficient treatment of depression-related disorders. Therefore, this current study proposes that the government, non-governmental agencies, and other philanthropists should collaborate to provide the needed resources to the hospitals and other medical facilities to help prevent and treat depression disorders. Additionally, this study suggests that low and middle-income nations with limited or scarce resources for treating and preventing depression disorders should employ internet-based interventional measures (Jiménez-Molina et al., 2019; Rojas et al., 2019). Thus, the government should adopt internet-based approaches perceived as more accessible and less expensive for adolescents. Further, pragmatic steps to bridge the treatment disparities among the populace should be initiated (Jiménez-Molina et al., 2019; Rojas et al., 2019). Finally, this research suggests that early interventional measures focused on subthreshold symptoms should be implemented for monitoring via digital technology to face-to-face pharmacotherapy or psychotherapy. This approach is perceived to the effectiveness and efficiency of health services delivery through the adoption of policy interventions to the particular needs of the individuals suffering from depression disorder.

## Data Availability Statement

The raw data supporting the conclusions of this article will be made available by the authors, without undue reservation.

## Author Contributions

QY: conceptualization and study design. ZW: formal analysis. ZL: data curation. XL: software. FO: writing and reviewing. XW: supervision. All authors contributed to the article and approved the submitted version.

## Conflict of Interest

The authors declare that the research was conducted in the absence of any commercial or financial relationships that could be construed as a potential conflict of interest.

## Publisher’s Note

All claims expressed in this article are solely those of the authors and do not necessarily represent those of their affiliated organizations, or those of the publisher, the editors and the reviewers. Any product that may be evaluated in this article, or claim that may be made by its manufacturer, is not guaranteed or endorsed by the publisher.
